# Effects of transdermal versus oral hormone replacement therapy in postmenopause: a systematic review

**DOI:** 10.1007/s00404-022-06647-5

**Published:** 2022-06-17

**Authors:** Marina Šprem Goldštajn, Mislav Mikuš, Filippo Alberto Ferrari, Mariachiara Bosco, Stefano Uccella, Marco Noventa, Peter Török, Sanja Terzic, Antonio Simone Laganà, Simone Garzon

**Affiliations:** 1 Department of Obstetrics and Gynecology, University Hospital Centre, Zagreb, Croatia; 2 School of Medicine, University of Zagreb, Zagreb, Croatia; 3 Department of Obstetrics and Gynecology, AOUI Verona, University of Verona, Verona, Italy; 4 Department of Women and Children’s Health, Clinic of Gynecology and Obstetrics, University of Padua, Padua, Italy; 5 Faculty of Medicine, Institute of Obstetrics and Gynecology, University of Debrecen, Debrecen, Hungary; 6 Department of Medicine, School of Medicine, Nazarbayev University, Nur-Sultan, 010000 Kazakhstan; 7 Unit of Gynecologic Oncology, ARNAS “Civico – Di Cristina – Benfratelli”, Department of Health Promotion, Mother and Child Care, Internal Medicine and Medical Specialties (PROMISE), University of Palermo, Palermo, Italy

**Keywords:** Hormone replacement therapy, Administration route, Postmenopausal, Venous thromboembolism, Metabolism, Cancer, Cardiovascular risk

## Abstract

**Purpose:**

To summarize available evidence comparing the transdermal and the oral administration routes of hormone replacement therapy (HRT) in postmenopausal women.

**Methods:**

We performed a systematic review of the literature on multiple databases between January 1990 and December 2021. We included randomized controlled trials and observational studies comparing the transdermal and oral administration routes of estrogens for HRT in postmenopausal women regarding at least one of the outcomes of interest: cardiovascular risk, venous thromboembolism (VTE), lipid metabolism, carbohydrate metabolism, bone mineral density (BMD), and risk of pre-malignant and malignant endometrial lesions, or breast cancer.

**Results:**

The systematic literature search identified a total of 1369 manuscripts, of which 51 were included. Most studies were observational and of good quality, whereas the majority of randomized controlled trials presented a high or medium risk of bias. Oral and transdermal administration routes are similar regarding BMD, glucose metabolism, and lipid profile improvements, as well as do not appear different regarding breast cancer, endometrial disease, and cardiovascular risk. Identified literature provides clear evidence only for the VTE risk, which is higher with the oral administration route.

**Conclusions:**

Available evidence comparing the transdermal and oral administration routes for HRT is limited and of low quality, recommending further investigations. VTE risk can be considered the clearest and strongest clinical difference between the two administration routes, supporting the transdermal HRT as safer than the oral administration route.

**Supplementary Information:**

The online version contains supplementary material available at 10.1007/s00404-022-06647-5.

## Introduction

Women’s life expectancy has increased by almost 10 years over the past half-century and is now approximately 78–86 years in most European countries [1, 2].Therefore, a woman can spend almost half of her life in peri- and postmenopause, with a consistent risk of developing a range of estrogen deficiency symptoms and diseases [3].

The transition to menopause is characterized by permanent cessation of ovarian function, leading to bothersome menopausal symptoms and long-term health consequences [4]. In this regard, hormone replacement therapy (HRT) has been proposed as a strategy to relieve menopause symptoms for years and conventionally includes both estrogen and progesterone [5]. Nevertheless, HRT use decreased dramatically after the results of the Women’s Health Initiative (WHI) trial in 2002 [6]. The WHI study was stopped due to increased myocardial infarction occurrence, thromboembolic events, and breast cancer cases in HRT users [6]. The HRT use dropped to 12% in 2004 and 5% in 2010 [7]. Nevertheless, the WHI trial was criticized for the presence of limitations and biases that should be considered to appropriately interpret study results, such as the inclusion of women aged 60–79 years [6].This crucial consideration pushes toward the foundations of the modern HRT, which consider the importance of starting HRT in the early years after menopause, introducing the concept of “time frame/window” of opportunity for the benefits of HRT [7, 8].

In this scenario, evaluating the best and maybe the safest administration route for HRT is of high relevance. Transdermal HRT is differently metabolized than the oral route, with a lower effective dose [9]. The skin metabolizes estradiol (E_2_) in a small part, and a reduced amount of hormone is required with lower serum estrone (E_1_) concentration, similar to premenopausal levels [10]. Based on differences between the oral and transdermal administration routes, the purpose of the present review was to summarize available evidence comparing the transdermal route with the oral administration of the estrogen component of the HRT in postmenopausal women. We focused on cardiovascular risk, venous thromboembolism (VTE), lipid metabolism, carbohydrate metabolism, bone mineral density (BMD), and risk of pre-malignant and malignant endometrial lesions and breast cancer.

## Materials and methods

### Sources and search strategy

A systematic literature search was conducted by two independent reviewers (M.Š.G. and M.M.) according to the Preferred Reporting Items for Systematic Reviews and Meta-Analyses (PRISMA) statement for transparent reporting of systematic reviews and meta-analyses [11]. The databases PubMed, clinicaltrials.gov, Scopus, and Web of Science were systematically searched for records from January 1990 to March 2021 using the combination of the medical terms “HRT”, “estrogen replacement”, “hormonal menopausal therapy”, “estrogen replacement therapy”, “menopausal therapy”, “menopausal hormone therapy”, “estrogentherapy”, and “estrogen replacement therapy.”

### Selection criteria

We included only studies reported in the English language. Allowed study designs were randomized and non-randomized controlled trials, observational prospective studies, and retrospective studies. The population of interest was postmenopausal women. Investigated interventions were oral and transdermal estrogen administration for HRT. The definition of transdermal estrogen application included gel, patch, or spray. Studies based on either estrogen monotherapy, combined-cyclic, or combined-continuous HRT were included, as well as studies investigating both natural, synthetic, or conjugated equine estrogens. Studies had to report regarding at least one of the following outcomes of interest: cardiovascular risk (acute coronary disease/myocardial infarction), VTE risk, variation of lipid profile values, alteration of carbohydrate metabolism, risk of pre-malignant and malignant endometrial lesions, risk of breast cancer, and variation in BMD.

### Study selection and data extraction

Titles and abstracts of identified studies were screened independently by two review authors (M.Š.G. and M.M.). The full text of the potentially eligible studies was retrieved and independently assessed for eligibility by two other review team members (F.A.F. and S.G.). Any disagreement over the eligibility of studies was resolved through discussion with a fifth author (A.S.L.). The reference lists of all identified studies were systematically revised to identify other eligible publications.

### Quality assessment

Two review authors (M.N. and P.T.) independently assessed the risk of bias of included randomized controlled trials according to the Cochrane risk-of-bias tool for evaluating the quality of randomized controlled trials (Rob 2.0) [12]. The following characteristics were considered: adequacy of randomization, allocation concealment, blinding of patients and outcome assessors, reporting of study withdrawals, the performance of an intention-to-treat analysis, and other potential biases.

The same team members assessed the methodological quality of non-randomized studies using the nine-star Newcastle Ottawa Scale (NOS) [13]. Each study was evaluated based on eight items, categorized into three broad perspectives including selection, comparability, and outcome for cohort studies or exposure for case–control studies. We considered studies with a score of 7 or greater as high quality. Disagreements between the review authors over the risk of bias were resolved by discussion with a third author (S.U.).

## Results

### Literature search

**Fig. 1:**
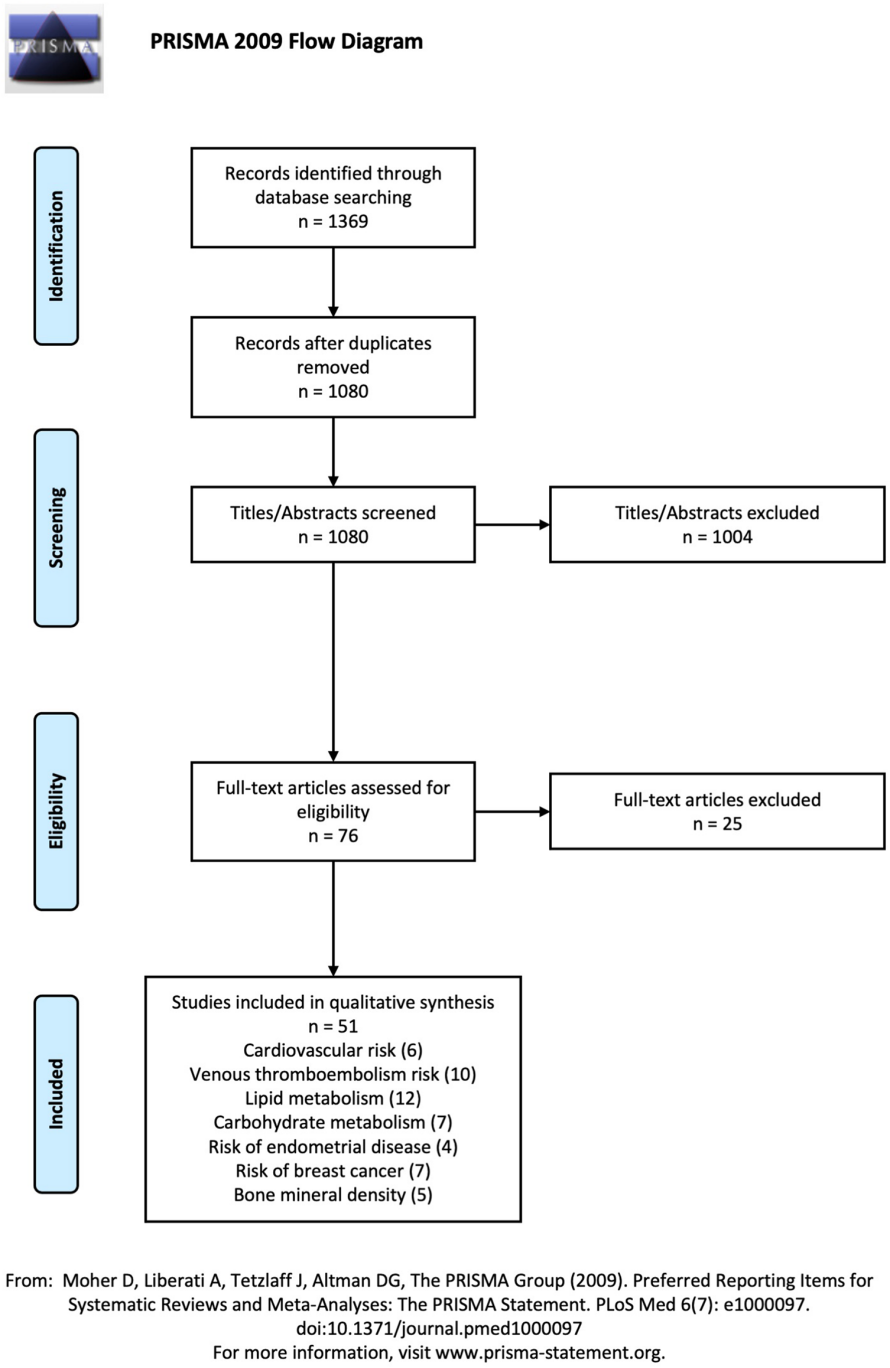
PRISMA flowchart of the study selection

The systematic literature search identified a total of 1369 manuscripts, of which 289 duplicate papers were removed. After title and abstract screening, 76 potentially relevant articles were identified and underwent full-text assessment for eligibility. Among these, 25 studies were excluded following inclusion and exclusion criteria. A total of 51 studies were finally included in the qualitative synthesis. The PRISMA flowchart summarizing study selection is reported in Fig. 1. Included manuscripts have been classified into seven groups according to the reported outcome: 6 studies were included in the “cardiovascular risk” group, 10 in the “VTE risk” group, 12 in the “lipid metabolism” group, 7 in the “carbohydrate metabolism” group, 5 in the “endometrial disease risk” group, 7 in the “breast cancer risk” group, and 5 were included in the “BMD” group. The main characteristics of the included studies are summarized in Tables 1–8.

**Table 1:** Cardiovascular risk: features of studies comparing oral and transdermal hormone replacement therapy

Author, year	Study design	Compared groups	Results
Varas-Lorenzo C et al. 2000	Case–control	Group 1: HRT Oral Transdermal Group 2: never-use HRT	*Medium estrogen dosage* O-CEE (OR 0.63; 95% CI 0.46–0.86) T-E_2_ (OR 0.62; 95% CI 0.37–1.06) Both route protective effect compared to Group 2 *Low estrogen dosage* (O-CEE < 0.625 mg, t-E2 0.025 mg) Not identified a protective effect or increasing risk
Chilvers ECD et al. 2003	Case–control	Group 1: HRT Oral Sticker Implant Group 2 never-use HRT	*Risk for non-fatal outcome of myocardial infarction* O-E_2_: OR 0.68 (0.49–0.95) T-E_2_: OR 1.70 (0.58–4.98) *Risk for a fatal outcome of myocardial infarction* O-E_2:_OR 0.40 (0.26–0.63) T-E_2_: OR 1.31 (0.47–3.68)
Hippisley-Cox J et al. 2003	Case–control	Group 1: HRT Group 2: never-use HRT	*Risk of coronary heart disease* O-E2: OR 1.27 (0.88–1.84) T-E2: OR 1.61 (0.76–3.39)* *The range for t-E2 is large due to the small number of subjects on this form of treatment
DeVries CS et al. 2006	Case–control	Group 1: HRT Oral estrogens Transdermal E_2_ Group 2: never-use HRT	*Risk of myocardial infarction* O-E_2_: OR 0.77 (0.66–0.90) T-E_2_: OR 0.66 (0.49–0.88)
Corrao G et al. 2007	Cohort study	Group 1: HRT > 3 years Oral estrogens Transdermal E_2_ Group 2: HRT < 6 months	*Overall risk of hospitalization due to ischemic heart disease* T- E_2_: RR 0.53 (0.34–0.82) O-E_2_: RR 1.15 (0.47–2.79) *Risk of hospitalization due to ischemic heart disease after prolonged use period (*> *3yrs)* O-E_2_: RR of 1.80 (0.66–4.88) T-E_2_: RR of 0.59 (0.33–1.05)
Lokkegaard E et al. 2008	Cohort study	Route of administration: Oral estrogens Transdermal E_2_ Estrogens only Combined	*Risk of myocardial infarction* O-E_2_ RR: 0.98 (0.67–1-12) - T-E_2_ RR of 0.62 (0.42–0.93) Combined HRT group: O-E_2_ RR of 1.08 (0.98–1.19); T-E_2_, RR of 0.95 (0.63–1-43); (p = 0.33) Vaginal estrogens RR of 0.56 (0.44–0.71)

*HRT* hormone replacement therapy, *RR* relative risk, *HR* hazard ratio, *OR* odds ratio, *O* oral, *T* transdermal, *CEE* conjugated equine estrogens

### Cardiovascular risk

Several studies investigated the effect of HRT (regardless of the route of administration) on the cardiovascular system, particularly affecting the coagulation cascade, inflammatory parameters, lipid composition, intima–media artery thickness, blood pressure, and atherosclerosis progression [14, 15]. Nevertheless, only four case–control studies and two cohort studies compared the risk of acute coronary disease (myocardial infarction) in women treated with oral or transdermal HRT [16–21]. Characteristics of the selected studies are reported in Table 1. Almost all studies agree with a beneficial effect of HRT, but none of the two routes of administration demonstrated a significant advantage, and heterogeneous results were globally reported. Moreover, none of these studies was designed to compare the two administration routes.

### Venous thromboembolism risk

VTE is a rare, but serious risk associated with HRT. Our systematic literature search identified ten studies comparing transdermal versus oral administration and their correlated risk with VTE events. Seven were case–control studies, of which three were multicenter, and three were cohort studies (Table 2).

**Table 2:** Venous thromboembolism risk: features of studies comparing oral and transdermal hormone replacement therapy

Author, year	Study type	Compared groups	Results
Daly et al. 1996	Case–control study	Group 1: HRT Oral Transdermal Implant Tibolone Group 2: never-use HRT	O-E_2_: OR of 4.6 (2.1–10.1) T-E_2_: OR of 2.0 (0.5–7.6) No significant difference between o-E_2_ and t-E_2_ No significant difference between high- and low-dose therapy No significant difference between non-opposing estrogens and combined estrogen–progesterone therapy
Perez Gutthann S et al. 1997	Case–control study	Group 1: HRT Oral Transdermal No HRT Group 2: never-use HRT	O-E_2_: OR 2.1 (1.3–3-6) T-E_2_: OR 2.1 (0.9–4.6) No significant difference between o-E_2_ and t-E_2_ No significant difference between high- and low-dose therapy
Scarabin PY et al. 2003	Case–control study	Group 1: HRT Oral Transdermal No HRT Group 2: never-use HRT	O-E_2_: RR 3.5 (95% CI 1.8–6.8) T-E_2_:RR 0.9 (95% 0.5–1.6) Transdermal administration is safer than oral route (RR 4.0, 95% CI 1.9–8.3)
Canonico M et al. 2007	Case–control study	Group 1: HRT Oral Transdermal Group 2: never-use HRT	O-E_2_: OR 4.2 (95% CI 1.5–11.6) T-E_2_:OR 0.9 (95% CI 0.4–2.1) Oral not transdermal estrogens were associated with increased thrombotic risk Micronized progesterone and pregnane not associated with an increased risk for VTE (OR, 0.7; 95% CI, 0.3 to 1.9 and OR, 0.9; 95% CI 0.4 to 2.3, respectively) Norpregnan: OR 3.9 (95% CI 1.5 to 10.0) Combination of transdermal estrogens and micronized progesterone is the safest choice
Canonico M et al. 2010	Cohort study	Group 1: HRT Oral Transdermal Group 2: never-use HRT	O-E_2_: HR 1.7 (95% CI 1.1–2.8) T-E_2_: HR 1.1(95% CI 0.8–1.8) Oral estrogens were associated with increased thrombotic risk Norpregnan had an increased risk for VTE, while other progesterone preparations did not show this effect
Renoux S et al. 2010	Case–control study	Group 1: HRT Oral Transdermal Group 2: never-use HRT	T-E_2_: RR 1.01 (95% CI 0.89–1.16)* T-E_2_ + progestogen: RR 0.96 (95% CI 0.77–1.20) O-E_2_: RR 1.49 (95% CI 1.37–1.63)** O-E_2_ + Progestogen: RR 1.54 (95% CI 1.44–1.65) *High dose did not increase the risk for VTE **High dose increased the risk for VTE
Sweetland S et al. 2012	Cohort study	Group 1: HRT Oral Transdermal Group 2: never-use HRT	O-E_2_: RR 1.42 (95% CI, 1.22–1.66) O-E_2_ + progestins: RR 2.07 (95% CI, 1.86 -2.32) T-E_2_: RR 0.82 (95% CI, 0.64–1.06)
Vinogradova et al. 2019	Case–control study	Group 1: cases of VTE Group 2: controls	E_2_ with MPA had the highest risk (OR 2.10, 1.92 to 2.31) E_2_ with dydrogesterone had the lowest risk (OR 1.18, 0.98 to 1.42) Estradiol lower risk than o-CEET-E_2_: OR 0.93 (95%-CI 0.87 to 1.01)
Bergendal et al. 2016	Case–control study	Group 1: HRT Group 2: never-use HRT	Current hormone therapy: OR 1.72 (95% CI 1.34–2.20) Estrogen + progestogen: OR 2.85 (95% CI 2.08–3.90) Estrogen only: OR 1.31 (95% CI 0.78–2.21) T-E_2_ + progestogen is not associated with higher risk
Simon et al. 2016	Cohort study	Group 1: HRT O-E_2_ (*N* = 316) T-E_2_ (*N* = 274) Group 2: never-use HRT	T-E_2_ users have significantly lower incidence of VTE events compared to o-E_2_cohort (RR 0.81; 95% CI 0.67–0.99)

*HRT* hormone replacement therapy, *VTE* venous thromboembolism, *RR* relative risk, *CI* confidence interval, *HR* hazard ratio; *o-CEE* *conjugated equine estrogens*

Only two studies found no difference between the two routes of administration [17, 22]. Conversely, other authors observed that transdermal HRT is a safer choice, especially in women at increased risk for VTE (Table 2) [22–31]. Renoux et al. reported an increased risk for VTE with high dosages of oral HRT, but not for the transdermal route, suggesting that the route of estrogen administration and concomitant progestogens type are important factors to define thrombotic risk [27]. Among different preparations, other authors suggested that the association of oral estrogen and medroxyprogesterone acetate seemed to correlate with the highest risk [29]. Noteworthy, Straczek et al. investigated the impact of the estrogen administration route on the association between VTE and the most common prothrombotic mutations of factor V of Leiden and prothrombin G20210A (Table 3) [32]. They observed that the oral administration route was associated with a higher increase in the OR for VTE than the transdermal route [32].

**Table 3:** Straczek et al., 2005; ESTHER study group—adjusted OR according to age, clinical center, and BMI for VTE

	No treatment (OR; 95% CI)	Oral estrogen (OR; 95% CI)	Transdermal estrogen (OR; 95% CI)
No mutations	1	4.1 (2.4–7.1)	1.2 (0.8–1.8)
One of the mutations*	4.1 (2.3–7.4)	25.5 (6.9–95)	4.4 (2.0–9.9)
Factor V Leiden mutation	3.2 (2.0–5.0)	6.3 (1.4–27.6)**	1.8 (0.5–6.3)**
Prothrombin mutation	4.8 (2.6–10.3)	/	1.5 (0.1–2.2)***

*OR* odds ratio, *CI* confidence interval, *BMI* body mass index, *VTE* venous thromboembolism

*compared to women with no mutation and no treatment **compared to women who have a factor V mutation and not used HRT; ***compared to women who have a prothrombin mutation and not used HRT

### Lipid metabolism

Our literature search found 12 studies comparing the effect of oral and transdermal HRT on lipid metabolism, as reported in Table 4: 11 randomized controlled trials and 1 cohort study. The 12 studies covered a wide time frame of 20 years and investigated a different combination of estrogens and progestogens. However, HRT reduced LDL values regardless of the administration route in all studies, although results provided by some of them using transdermal estrogens have not demonstrated a statistically significant decrease in LDL concentrations [33–35]. Additionally, oral replacement therapy was demonstrated to increase the HDL and triglycerides concentration. Conversely, transdermal therapy had no significant effect on HDL levels and most studies highlighted a significant reduction in triglyceride concentration [36–39].

**Table 4:** Lipid metabolism: features of studies comparing oral and transdermal hormone replacement therapy

Author, year	Study type		Compared groups	Results
Perrone G et al. 1996	Randomized controlled trial		Group 1 (*N* = 14) t-E_2_ 50 mcg + MPA 10 mg/day Group 2 (*N* = 14) o-E_2_ 0.625 mg/day + MPA 10 mg/day Group 3: never-use HRT (*N* = 14)	Total cholesterol and LDL cholesterol decreased after 6 months in both reported groups Small variations of HDL cholesterol and triglycerides were reported
Adami S et al. 1993	Randomized controlled trial		Group 1 o-E_2_ 0.625 mg/MPA 10 mg/12 days Group 2 (N = 27): t-E_2_ patch 0.05 mg/MPA 10 mg/12 days Group 3: never-use HRT	LDL diminished with estrogen replacement therapy HDL diminished with t-E_2_ and slightly increase with O-E_2_ Triglycerides diminished with t-E_2_ and slightly increased with O-E_2_
Whitcroft SI et al. 1994	Randomized controlled trial		Group 1 o-CEE 0.625 mg/dinorgestrol 0.15 mg/12 days Group 2 t-E_2_ patch 0.05 mg/NETA 0.25 mg/14 days Group 3: never-use HRT	o-CEE Total cholesterol decreased by 12.1% (*p* < 0.001) LDL levels decreased by 14.2% (*p* < 0.001) HDL decreased by 7.8% (*p* < 0.05) Triglycerides decreased by 2.5% (*p* < 0.05) and t-E_2_-16.4% (*p* < 0.01) T-E_2_ Total cholesterol decreased by 8.4% (*p* < 0.001) LDL levels decreased by 6.6% (*p* < 0.01) HDL decreased by 10.7%, (*p* < 0.001) Triglycerides decreased by 16.4% (*p* < 0.01) *The potentially beneficial effects of estrogen–progestin therapy on serum total and LDL cholesterol and on triglycerides were maintained over 3 years*
Spencer C et al. 1999	Randomized controlled trial		Group 1 o-E_2_ 2 mg/1 mg/NETA 1 mg Group 2 t-E_2_patch 0.05 mg/NETA 1 mg	O-E_2_ Total cholesterol decreased by 7% (*p* < 0.001) LDL levels increased by 3% (*p* < 0.001) HDL decreased by 3% (*p* < 0.05) Triglycerides increased by 9.4% (*p* < 0.05) T-E_2_ Total cholesterol decreased by 4% (*p* < 0.001) HDL decreased by 6%, (*p* < 0.001) Triglycerides decreased by 19% (*p* < 0.05)
Erneus M et al. 2001	Randomized controlled trial		Group 1 o-CEE 0.625 mg/MPA 2.5 mg Group 2 t-E_2_patch0.05 mg/MPA 2.5 mg	o-CEE Total cholesterol decreased by 1,9% (*p* < 0.001) after 1 year and by14.7% after 2 years LDL levels increased by 3% (*p* < 0.001) HDL decreased by 10.2% (*p* < 0.05) and by 31.4% after 2 years Triglycerides increased by 9.4% (*p* < 0.05) T-E_2_ Total cholesterol decreased by 6.2% (*p* < 0.001) after 1 year and 18% after 2 years HDL decreased by 13.5%, (*p* < 0.001) after 1 year and 33.6% after 2 years Triglycerides decreased by 33.7% (*p* < 0.05)
Araujo DA et al. 2002	Randomized controlled trial		Group 1 o-CEE 0.625 mg/micronized progesterone 300 mg/12 days Group 2 t-E_2_patch 0.05 mg/micronized P4 300 mg/12 days	o-CEE HDL and triglycerides significantly increased (9% and 20.7%, *p* = 0.04) Total cholesterol and LDL values did not change T-E_2_ Not statistically significant changes in lipid composition
Wakatsuki A et al. 2002	Randomized controlled trial		Group 1 o-CEE 0.625 mg Group 2 t-E2 patch 0.05 mg Group 3: never-use HRT	O-E_2_ Total cholesterol decreased LDL lower after treatment HDL significantly increased Triglycerides higher after treatment T-E_2_ Total cholesterol decreased Triglycerides significantly decreased HDL values did not change with treatment *The use of transdermal estrogen, but not oral, leads to larger LDL particles more resistant to oxidation, preserving the estrogen’s antioxidizing effect*
Nanda S et al. 2003	Randomized controlled trial		Group 1: HRT o-CEE 0.625 mg t-E_2_ patch 0.05 mg Group 2: never-use HRT	O-E_2_ Total cholesterol decreased by 7% (*p* < 0.001) after 7 months LDL levels decreased by 22% after 6 months Triglycerides increased by 8% (*p* < 0.01) T-E_2_ Total cholesterol decreased by 2% (*p* < 0.001) after 7 months LDL levels decreased by 16% after 6 months Triglycerides decreased by 6% (*p* < 0.05)
Sanada M et al. 2004	Randomized controlled trial		Group 1 o-CEE 0.625 mg/MPA 2.5 mg Group 2: after 12 months of o-E_2_ t-E_2_ patch 0.05 mg/MPA 2.5 mg	O-E_2_ Total cholesterol decreased by 1,9% (*p* < 0.001) after 1 year and by14.7% after 2 years Ratio of LDL and Apo-B decreased by 12.8%, (*p* < 0.05) HDL decreased by 2,6% (*p* < 0.05) Triglycerides increased by 78% (*p* < 0.05) T-E_2_ Total cholesterol decreased by 6.2% (*p* < 0.001) after 1 year and 18% after 2 years Ratio of LDL and Apo-B increased significantly (*p* < 0.05) LDL increased 4.8% HDL decreased by and additional 3.9%, Triglycerides decreased by 51% (*p* < 0.05)
Shakir YA et al. 2004	Randomized controlled trial		Group 1 0.05 mg/sequential NETA 0.25 mg 15–28. day Group 2 Oral E_2_ 2 mg/1 mg/continuous NETA 1 mg Group 3 Oral E_2_ 0.05 mg/sequential NETA 1 mg 23.-28.day	Total cholesterol higher with t-E_2_ compared to both o-E_2_ regimens (5.9 vs 5.68, *p* < 0.05) LDL no statistically significant differences in reported groups HDL no statistically significant differences in reported groups Triglycerides no statistically significant differences in reported groups
Vrablik M et al., 2008	Randomized controlled trial		Group 1 o-E_2_ 2 mg Group 2 t-E_2_ patch 0.05 mg	O-E_2_ Total cholesterol decreased by 4% (*p* < 0.01) LDL levels decreased by 19% (*p* < 0.001) HDL increased by 10.5% (*p* < 0.01) and by 31.4% after 2 years Triglycerides increased by 14% (*p* < 0.01) T-E_2_ Total cholesterol values did not change LDL levels decreased by 3,2% (*p* < 0.001) HDL increased by 5,2%, (*p* < 0.001) after 1 year and 33.6% after 2 years Triglycerides, no significant changes The atherogenic plasma index significantly reduced relative to o-E_2_ (-0.17 vs -0.23, *P* = 0.023)
Lee JY et al., 2015	Cohort study		Group 1 o-CEE 0.625 mg/micronized P4200 mg Group 2 t-E_2_ 0.1% 1.5 mg/micronized P4 200 mg	O-E_2_ Decreased LDL (*P* = 0.001) and elevated triglycerides (*P* = 0.007) and HDL (*P* = 0.001) T-E_2_ Decreased LDL and increase of triglycerides and HDL, although statistically insignificant. Triglycerides remained unchanged

*HRT* hormone replacement therapy, *o-CEE* *conjugated equine estrogens*, *LDL* low-density lipoprotein, *HDL* high-density lipoprotein, *MPA* medroxyprogesterone acetate, *NETA* norethindrone acetate

### Carbohydrate metabolism

Seven studies reporting on carbohydrate metabolism were identified, among which only one randomized controlled trial was found [40–46] (Table 5).The only study that directly compared the impact of oral and transdermal HRT on diabetes risk was the French prospective E3N study. The authors reported a higher reduction of diabetes risk in oral HRT users compared to the transdermal arm, although both administration routes reduced the risk as compared to women without HRT and were associated with a reduction in glycated hemoglobin concentration [41]. However, the study did not consider the type of progestogens that was used in combination with the estrogen, and the results could reflect the influence of individual progestogens rather than the estrogen administration route. Notably, Shakir et al. reported a negative impact on glucose tolerance and insulin resistance by medroxyprogesterone acetate and levonorgestrel [40]. Another study was not able to show significant differences between the progestogens used in the HRT [41].

**Table 5:** Carbohydrate metabolism: features of studies comparing oral and transdermal hormone replacement therapy

Author, year	Study type	Compared groups	Results
Godsland IF et al. 1993	Randomized controlled trial	Group 1 o-CEE 0.625 mg/LNG 0.075 mg 12 days Group 2 t- E_2_ 0.05 mg/NETA 0.25 mg 14 days Group 3 Control group	O-estrogen determined a deterioration in glucose tolerance (*p* = 0.05) O-estrogens caused a decrease in insulin resistance during the combined NETA treatment phase, higher compared to t-E_2_ (*p* < 0.05) t-E_2_ showed no changes reported in insulin values and insulin sensitivity
OʼSullivan A et al. 1998	Randomized controlled trial	Group 1 o-CEE 1.25 mg / MPA 10 mg 12 days Group 2 t-E_2_patch 0.01 mg / MPA 10 mg 12 days	O-E_2_ Lower IGF-1 compared to t-E_2_ (*p* < 0.01) Reduction in lipid oxidation measured 30–60 min post-prandially (*p* < 0.01) Increase in carbohydrates oxidation (*p* < 0.05) Increase the proportion of fat tissue (5.2%) and decreased proportion of non-fat tissue (2%) compared to t-E_2_ (*P* < 0.01) No changes in BMI in the both route of administration
Karjalainen A et al. 2001	Randomized controlled trial	Group 1 o-estrogen valereate 2 mg Group 2 t-E_2_ beta-oestradiol gel 1 mg	Both o-E_2_ and t-E_2_ reduced in HbA1c levels (*p* < 0.05) The OGTT and postprandial insulin levels did not change significantly C-peptide levels increased by 8% in both treated groups O-E_2_ decreased IGF-1 values and increased GH values (*p* < 0.05)
dos Reis CM et al. 2003	Randomized controlled trial	Group 1 o-CEE 0.625 mg Group 2 t-E_2_ patch 0.05 mg Group 3 Control group	No difference in body weight, visceral fat, BMI, o-E_2_ or t-E_2_ Statistical decrease in IGF-I (*p* < 0.05) and increase in GH values (*p* < 0.05) with o-E_2_. No significant changes in t-E_2_ group Increase in fat tissue content with o-E_2_ (12%, *p* < 0.05). No significant changes with t-E_2_ Proportion of non-fat component increased with t-E_2_ (3%, *p* < 0.05) and decreased with o-E_2_ (7%, *p* < 0.05) Fat oxidation decreased and carbohydrate oxidation increased with o-E_2._Opposite changes recorded with t-E_2_ (*p* < 0.05)
Shakir YA et al. 2004	Randomized controlled trial	Group 1 o-E_2_ 1 mg/MPA 10 mg 14 days Group 2 t-E_2_ product 0.05 mg/MPA 10 mg 14 days	The lowest number of women with IGT was found in the group on t-E_2_ compared to the groups on o-E_2_ regimen (16.4% vs 31%, *P* = 0.001) IGT is more common in the continuous o-E_2_ regimen (31.8%) compared to the sequential o-E_2_ and t-E_2_ (18.5%, *P* = 0.002)
Chu CM et al. 2006	Randomized controlled trial	Group 1 o-E_2_ 1 mg/MPA 10 mg/14 days Group 2 t-E_2_ preparation 0.05 mg/MPA 10 mg 14 days	*o-E*_*2*_* in patients with IR determined a deterioration of the IR markers:* Ratio of fasting glucose to insulin decreased (*p* < 0.01) Insulin concentration increased (*p* < 0.01) HOMA index increased (*p* < 0.05) *T-E*_*2*_* in patients with IR did not determine significant changes in IR markers:* Ratio of fasting glucose to insulin decreased (*p* < 0.05)
De Lauzon-Guillain B et al. 2009—E3N Study	Cohort study	Group 1 o-CEE Group 2 t-estrogen Group 3 Never-use HRT	Lower risk for DM was observed in HRT users (HR 0.82; 95% CI 0.72–0.93) compared to never-users HRT Adjustment for BMI during follow-up did not change the association O-E_2_ reduced the risk of DM compared to t-E_2_ (HR = 0.61 vs 0.78 *P* = 0.031) Subjects on HRT had a higher BMI increase per year than those controls (*p* < 0.001)

*IGF-1* insuline-like growth factor 1, *OGTT* oral glucose-tolerant test, *IGT* impairment glucose tolerance, *IR* insuline resistance, *DM* diabetes mellitus, *BMI* body mass index, *HRT* hormone replacment therapy, *HR* hazard ratio, *o-CEE* *conjugated equine estrogens*

Overall, according to the published results, both oral and transdermal administration routes reduce insulin resistance, with a more evident effect of the oral administration route in non-diabetic women.

### Risk of pre-malignant and malignant lesions of the endometrium

Concerning the risk of developing endometrial hyperplasia or endometrial cancer, a small number of studies were designed to compare transdermal and oral estrogenfor HRT (Table 6) [47–50]. Four randomized controlled trials were identified and included in our results. The authors did not demonstrate an increased risk of malignant and pre-malignant endometrial lesions with transdermal or oral administration in combined therapy or differences between them. Different authors reported a risk of endometrial hyperplasia and cancer with the transdermal route comparable to or even lower than those associated with the oral administration route. Vaginal bleeding was reduced with longer use, and amenorrhea was achieved in an equal percentage of women with oral and transdermal HRT.

**Table 6:** Risk of pre-malignant and malignant lesions of the endometrium: features of studies comparing oral and transdermal hormone replacement therapy

Author, year	Study type	Compared groups	Results
Mattsson LA et al. 1999	Randomized controlled trial	Group 1 o-E_2_ 2 mg/NETA 1 mg (*N* = 108) Group 2 t-E_2_ patch 0.05 mg/NETA 0.25 mg (*N* = 94) Group 3 t-E_2_ patch 0.025 mg/NETA 0.125 mg (*N* = 116)	2% of cases of endometrial hyperplasia One case of simple hyperplasia in the o-E_2_ and t-E_2_ 0.025 mg group From 9 to 12th month, amenorrhea occurred in 85% int-E_2_ 0.025 mg group, 65% in t-E_2_ 0.05 mg group, and 79% in users on oral therapy
Sendag F al. 2001	Randomized controlled trial	Group 1 o-CEE 0.625 mg/MPA 10 mg 10 days Group 2 t-E_2_ patch 0.05 mg/NETA 0.25 mg 14 days	Atrophic endometrium in 21.2% and 17.1% of women using o-CEE and t-E_2_ Secretory endometrium in 62.2% and 65.7% of women using o-CEE and t-E_2_ Proliferative endometrium in 13.5% and 14.3% of women using o-CEE and t-E_2_ Endometrial hyperplasia in 2,7% and 2,9% of woman using o-CEE and t-E_2_
Samsioe G et al. 2007	Randomized controlled trial	Group 1 o-E_2_ 1 mg/NETA 0.5 mg daily Group 2 t-E_2_ patch 0.025 mg/NETA 0.125 mg 2x/week	No case of endometrial hyperplasia or cancer was reported Endometrial thickness ≥ 5 mm: 10.5% of women on t-E_2_ and 11.5% on o-E_2_ Endometrial polyps: 1% on t-E_2_ and 1.5% on o-E_2_ Intermenstrual bleeding was reduced by 98% for the t-E_2_ group and 99% for the o-E_2_ group
Russu M et al. 2015	Randomized controlled trial	Group 1 o-E_2_ valerate 2 mg/micronized E_2_ 2 mg/dydrogesterone Group 2: non oral t- E_2_ gel 1 g/vaginally micronized P4 200 mg or t-E_2_ patch 0.025 mg/MPA 10 mg/5 mg	Proliferative endometrium less frequent in non-oral versus o-E_2_ group (*p* < 0.01, 3.2% vs 9% after 12 months, 4.5% vs 10.8% after 24 months); Secretory endometrium more frequent in non-oral versus o-E_2_ group (*p* < 0.01, 80.6% vs 63.6% after 12 months, 51.6% vs 36.7% after 24 months) Atrophic endometrium more frequent in o-E_2_ vs non-oral group (*p* < 0.01, 9.81% vs 9.35% after 12 months, 40.5% vs 16.4% after 24 months)

*HRT* hormone replacement therapy, *o-CEE* *conjugated equine estrogens*, *NETA* norethisterone acetate, *RR* relative risk, *CI* confidence interval

### Risk of breast cancer

Our literature search identified six cohort studies and one case–control study comparing the oral and transdermal HRT effects on breast cancer risk (Table 7) [16, 51–56]. The UK Million Women Study [51] was the first study investigating the effect of the estrogen administration route on breast cancer risk and no differences were found between oral and transdermal administration. Subsequently, in 2006, Lyytinnen et al. achieved the same conclusions after comparing oral and transdermal HRT with high, medium, and low estrogen doses and equal treatment lengths [53]. The same authors compared the effect of combined oral and transdermal HRT with progestogens supplementation in 2009 [54]. Once more, no significant differences were found between oral and transdermal estrogen administration. Furthermore, 5 years after cessation of therapy, the risk of breast cancer was the same for non-users [54]. Similarly, the data of the French E3N cohort study reported an increased risk of breast cancer among women receiving HRT without differences between oral and transdermal administration routes, although authors suggested preferring micronized progesterone to synthetic progestins [52, 55]. In a study based on the British family physicians database, Opatrny et al. [56] reported an increased risk of breast cancer among HRT users of oral estrogens; however, the same was not observed in users of transdermal products. Nevertheless, intervals were broad, and the overlap with the results from the oral group did not make a statistically significant difference between the two types of HRT administration routes.

**Table 7:** Risk of breast cancer: results of studies comparing oral and transdermal administration of hormone replacement therapy

Author, year	Study type	Compared groups	Results
Beral V et al. 2003	Cohort study	Group 1 Oral Transdermal Group 2 Controls	HRT increased the risk of breast cancer compared to controls (RR 1.66 [95% CI 1.58–1.75], *p* < 0.0001) HRT increased mortality compared to controls (RR 1.22 [1.00–1.48], *p* = 0.05) Risk for breast cancer slightly higher on oral HRT group compared to as transdermal HRT, but difference was statistically insignificant [RR 1.32 (1.21–1.45)] vs [RR 1.24 (1.11–1.39)] Oral combined therapy had a higher risk compared with estrogen-only preparation (RR 2.00 vs 1.30, *p* < 0.0001)
Fournier A et al. 2005	Cohort study	Group 1 Oral Group 2 Transdermal Group 3: controls	HRT increased risk of breast cancer compared to controls (RR 1.22; 1–1-1.4) Oral HRT with RR 1.5 (1.1–1.9), transdermal HRT route RR 1.4 (1.2–1.7, *p* < 0.001), with statistically insignificant difference between the two routes of administration Estrogen-only therapy RR 1.1 (0,8–1,6); Estrogen combined with oral progestogens RR 1.3 (1.1–1.5) The risk is higher with HRT containing synthetic preogestins than micronized progesterone
Lyytinen H et al. 2006	Cohort study	Group 1 Oral E_2_ Group 2 Transdermal E_2_ Group 3 Vaginal estrogens	HRT < 5 yr is not associated with an increased risk for breast cancer (OR 0.93; 0.80–1.04) HRT > 5 yr associated with an increased risk (OR 1.44; 1.29–1.59) Oral and transdermal preparations similar risk for breast cancer Vaginal estrogen not associated with an increased risk Low doses of E_2_: oral [OR 1.15 (0.71–1.75)]; transdermal [1.60 (0.77–2.95)] Medium doses of E_2_: oral [ OR 1.38 (0.84–2.12)]; transdermal [1.32 (1.12–1.64)] High doses of E_2_: oral [1.49 (1.25–1.75)]; transdermal [1.44 (0.88- 2.22)]
Fournier A et al. 2008 – E3N	Cohort study	Group 1 Oral Group 2 Transdermal	Oral combined HRT [RR of 1.31 (0.76–2.29)]; transdermal combined HRT [1.28 (0.98–1.69)] Oral [OR 0.77 (0.36–1.62)] and transdermal [1.18 (0.95–1.48)] E_2_ combined with dydrogesterone had no increased risk Oral [OR 2.74 (1.42–5.29)] and transdermal [2.03 (1.39–2.97) E_2_ combined with MPA had increased risk Oral [RR 2.02 (1.00–4.06)] and transdermal E_2_ [RR 1.48 (1.05–2.09)]combined with CMA had increased risk Oral [RR 1.62 (0.94–2.82)] and transdermal E_2_ [RR 1.52 (1.19–1.96)] combined with promegestone had increased risk Oral [RR 1.10 (0.55–2.21)] and transdermal E_2_[1.60 (1.28–2.01)] combined with NMA had increased risk
Opatrny S et al. 2008	Case–control study	Group 1 Oral opposed estrogens Group 2 Transdermal opposed estrogens Group 3 Non-opposed HRT Group 4: controls	Oral opposed estrogens HRT had an increased risk [OR 1.38(1.27–1.48)] Transdermal opposed estrogens HRT had not an increased risk [OR 1.08 (0.81–1.43)] No difference between sequential or continuous regimen of combined HRT Non-opposed HRT had no increased risk for breast cancer [RR 0.97 (0.86–1.09)]
Corrao G et al. 2008	Cohort study	Group 1: HRT > 2 years Oral Transdermal Group 2: HRT < 6 months Oral Transdermal	HRT > 2 years higher risk than HRT therapy < 6 months [RR 1.34(1.13–1.58)] Oral HRT: RR 2.14(1.43–3.21) Transdermal HRT: RR 1.27(1.07–1.51)
Lyytinen H et al. 2009	Cohort study	Group 1 Oral Group 2 Transdermal	Up to 3 yr oral and transdermal HRT did not increase risk for breast cancer (RR 1.05; 0.99–1.12, 931 cases vs RR 0.99: 0.79–1.23, 82 cases) Between 3rd and 5th: oral [RR 1.27 (1.15–1-39)] transdermal [RR 1.38 (1.01–1.85)] After 5 years: oral [RR 1.81 (1.73–1.89)] transdermal [RR 1.60 (1.11–2.23)] Preparations with NETA had a higher risk for breast cancer compared to preparations with dydrogesterone and MPA

*HRT* hormone replacement therapy, *RR* relative risk, *OR* odds ratio, *NETA* norethisterone acetate, *CMA* chlormadinone acetate, *MPA* medroxyrogeterone acetate, *NMA* *nomegestrol* acetate

### Effect on bone mineral density

Four randomized prospective studies and one retrospective case–control study were identified (Table 8) [57–62]. Both oral and transdermal administration routes demonstrated a positive effect on BMD values. Early start and higher doses showed a greater effect regardless of the administration route. In the most recent study by Kim et al., the authors reported a positive increasing trend after 24 months of HRT as compared to baseline and 12-month BMD values.

**Table 8:** Effects on bone mineral density: results of studies comparing oral and transdermal administration of hormone replacement therapy

Author, year	Study type	Compared groups	Results
Stevenson JC et al. 1990	Randomized controlled trial	Group 1 o-CEE 0.625 mg/norgestrel 1.5 mg/12 days Group 2 t-E_2_ patch 0.05 mg/noresetisteron acetate 0.25 mg 14 days Group 3 Placebo	Bone degradation was statistically significantly reduced in both HRT administration routes BMD increased, with no significant difference between Group 1 and 2
Palacios S et al. 1994	Randomized controlled trial	Group 1 o-CEE 0.625 mg Group 2 t-E_2_ patch 1.5 mg Group 3 Placebo	BMD increased with t-E_2_ by 1.7% after 12 months, 5.6% after 24 months, and 4.7% after 36 months (*p* < 0.001) BMD increased with o-CEE by 3.5% after 12 months and 4% after 24 months (*p* < 0.001) BMD loss with placebo was 6.6% after 12 months and 9.1% after 24 months (*p* < 0.001)
Cetinkaya MB et al. 2002	Randomized controlled trial	Group 1 o-CEE Group 2 o-CEE/MPA Group 3 t-E_2_ patch	After 24 months BMD increased in all treated subjects The increase in BMD was: t-E_2_ 2.35% (± 13.19), unopposed o-E_2_ 1.37% (± 8.39), combined o-E_2_ 4.08% (± 19.39)
Davas I et al. 2003	Randomized controlled trial	Group 1 o-CEE 0.625 mg/MPA 5 mg Group 2 t-E_2_ patch 0.05 mg/MPA 5 mg Group 3 o-CEE 0.625 mg/MPA 5 mg and alendronate Group 4 t-E_2_ patch 0.05 mg/MPA 5 mg and ale dronate	BMD lumbar spine increase registered in all groups For patients with osteopenia, o-CEE increased BMD by 3.3%, and t-E_2_ increased BMD by 2.9% For patients with osteoporosis o-CEE increased BMD by 7.3% and t-E_2_ increased BMD by 6.6% Hormone therapy plus alendronate increased the BMD more in the osteoporotic group than in the osteopenic group (p = 0.001)
Kim H et al. 2014	Case–control study	Group 1 o-CEE 0.625 mg Group 2 t-E_2_ patch 1.5 mg or 0.1% E_2_ gel 1.5 mg E_2_ Group 3 Placebo	After 12 months, lumbar spine BMD increased in treated groups by 3.4% with no statistically difference in Group 1 and 2 After 12 months, hip BMD increased by 2.1% with o-E_2_ and by 3.9% with t-E_2_ After 24 months, lumbar spine BMD increased by 4.8% with o-E_2_ and by 4.9% with t-E_2_ After 24 months, hip BMD increased by 3.5% with o-E_2_ and by 4.2% with t-E_2_ No difference between patch and gel on BMD values No effect on BMD values after addition of progesterone

*BMD* bone mineral density, *o-CEE* *conjugated equine estrogens*,* MPA medroxyprogesterone acetate*

### Risk of bias assessment

Risk of bias assessment for randomized controlled trials was performed according to the Cochrane risk-of-bias tool and summarized in Supplementary Table 1. Overall, the risk of bias was high for most of the identified randomized controlled trials, followed by unclear risk. Only one randomized controlled trial was classified as having a low risk of bias [44]. Conversely, the risk of bias assessment for observational studies reported a quality score equal to or higher than 6 in all included studies (Supplementary Table 2).

## Discussion

Transdermal estrogen preparations are considered as effective for treating menopausal symptoms [63] as the oral administration route [64]. However, the transdermal administration route has different pharmacodynamics as compared to oral administration [65], determining possible different safety profiles and impacts on global women's health [64]. In our systematic review of the literature, we observed relevant differences between the two administration routes that highlight the need to further characterize the similarities and differences between these two administration options.

Nowadays, HRT is not recommended for the primary or secondary prevention of cardiovascular disease [66, 67]. This position is primarily based on the WHI trial findings, which raised concerns about an increased risk of acute coronary disease and breast cancer. However, the re-analysis of data from the WHI estrogen-only [68] arm study has demonstrated that the impact of HRT on acute coronary disease risk is related to the woman's age at the beginning of HRT administration. HRT decreased by 44% the acute coronary disease risk in the group of women younger than 60 years. Conversely, the study did not report benefits in the group older than 60 years for the acute coronary disease, but showed an increased risk for stroke. These observations suggested that an early start after menopause is required to achieve benefits on cardiovascular risk. Conversely, when HRT is started regardless of age, no benefits are found for primary or secondary prevention of all-cause mortality, cardiovascular-related death, non-fatal heart infarction, or need for revascularization [69]. Regarding possible differences in cardiovascular risk between the oral and transdermal administration routes, we did not identify studies suggesting differences. Most studies included in our review highlighted a possible beneficial effect of HRT, but none of the two administration routes demonstrated significant advantages over the other [17–19]. Consistently, cardiological societies prefer transdermal therapy based on other elements of the safety profile instead of a demonstrated higher efficacy in improving cardiovascular risk. Our systematic review highlights a lack of evidence comparing the incidence of cardiovascular disease between the oral and transdermal administration routes, recommending further investigation [65–67].

Regarding VTE, all identified studies are consistent in reporting the transdermal administration route being safer than oral HRT. The avoidance of the first hepatic passage is the main reason explaining the absent increase of procoagulant factors with transdermal products. Three previously published meta-analyses confirmed these findings [70–72]. A 2008 meta-analysis reported an OR for VTE of 2.5 (95% CI 1.9–3.4), and 1.2 (95% CI 0.9–1.7), respectively, for oral and transdermal HRT compared to never users [71]. Moreover, Mohammed et al. demonstrated a higher relative risk of VTE in the oral estrogen group compared to transdermal estrogen [72], consistently with the conclusion of the meta-analysis by Rovinski et al. [70]. Noteworthy, in women with prothrombotic mutation, oral HRT leads to a 25-fold increased risk for VTE compared to non-users [25], versus a lower fourfold increased risk for VTE determined by transdermal estrogens [32]. Based on identified studies, the transdermal administration route of estrogens appears the preferred choice in terms of VTE risk.

After menopause, estrogen reduction causes a pro-atherogenic shift of lipid-lipoprotein profile with an increase in total cholesterol, LDL, lipoprotein-a, triglycerides, and a reduction of HDL levels [73]. In this regard, most of the included studies, the majority randomized controlled trials, did not observe differences between the two administration routes in terms of lipid–lipoprotein profile improvement. However, our systematic review suggests possible differences in lipid–lipoprotein profile changes between the two administration routes; some studies observed a higher improvement of triglyceride levels with the transdermal administration route and a higher impact on cholesterol metabolism with oral HRT.

Menopause is characterized by weight gain, decreased energy expenditure, loss of lean body mass, and an increase in the perivisceral and total amount of fat [74]. These changes have been related to the influence of estrogens on the growth hormone/insulin-like growth factor-I (GH/IGF-I) axis, decreasing IGF-I and increasing GH levels [75, 76]. GH and IGF-I play a pivotal role in body composition and in resting energy expenditure and finally influence glucose metabolism [77, 78]. Consistently, HRT was reported to significantly reduce the risk of diabetes and insulin resistance, without clear differences between oral and transdermal administration routes. Indeed, only one study directly compared the two administration routes [41], suggesting a higher reduction of diabetes risk in oral HRT users compared to transdermal ones. Nevertheless, further evidence is required to potentially recommend one administration route over the other based on the improvements in glucose metabolisms.

Concerning the risk of invasive breast cancer, literature reported a higher incidence among HRT users than never users. A little risk was demonstrated for a period shorter than 6 months [16], with progressively increasing risk for longer periods, although its magnitude has been found to vary across studies and a safe cutoff for HRT length has not been demonstrated. Moreover, although a higher risk was demonstrated for current users than past users, some studies showed that risk persists after HRT cessation [79]. Concerning the effect on breast cancer risk provided by the administration route, studies included in our systematic review did not show a different risk between the transdermal and oral administration routes, although the E_1_–E_2_ ratio is fivefold higher with oral E_2_ than in physiological conditions or with the transdermal route [55, 56]. Nonetheless, the findings of the included studies are not able to exclude a different risk. Therefore, further investigation is needed to clarify whether the transdermal route carries a lower risk or not [80]. In this regard, other administration routes, such as topical vaginal estrogen preparations, seemed to lead to lower risk, further reducing the systemic exposure [53, 81]. Finally, any future study must address differences in progestogens. Breast cancer risks with combined HRT did not appear to differ based on the progestogen; however, some studies suggest a higher risk with norethisterone acetate and a lower risk for dydrogesterone [54].

Endometrial cancer is the most common gynecological cancer in developed countries [82]. More than 90% of cases of endometrial cancer occur in women older than 50 years of age, with a median age at diagnosis of 63 years [83]. Based on the results of our systematic review, the risk of endometrial hyperplasia and endometrial cancer with transdermal HRT is equal to or smaller than with oral HRT [49, 50]. Moreover, the proportion of patients reporting amenorrhea did not differ between the two administration routes [50]. Notably, our systematic review confirms that the main risk factor for endometrial cancer, regardless of the administration route, is the use of only estrogen replacement therapy in women with the uterus.

Estrogen deficiency in menopause determines an accelerated bone loss [84]. Osteoporosis affects one-third of women aged over 50 years and is associated with increased social costs, mortality, and worse quality of life [85]. Both the uncombined and combined HRT arms of the WHI study showed a significant increase in BMD and a reduction of hip fractures than controls [85]. Thus, HRT could be considered the first-line therapy for maintenance of BMD in postmenopausal women under the age of 60 years or within 10 years after menopause, as indicated in international guidelines [86–89]. Meanwhile, after the age of 60 years, a joint Global Consensus Statement in 2016 stated that HRT is a second-line therapy for preventing fractures [90]. In this scenario, our findings showed an osteoprotective effect, with increased BMD, both with oral and transdermal routes [58, 61, 62]. Therefore, BMD prevention does not appear to guide the choice of the HRT administration route.

### Strengths and limitations

The strength of this study lies in the methodological approach of the comprehensive literature search, the following of The Preferred Reporting Items for Systematic Reviews and Meta-analyses (PRISMA), and the quality of the studies assessment. Conversely, our conclusions are limited by the disadvantage of most studies, which are based on small numbers of enrolled subjects, and by the limited number of studies comparing the two administration routes. Notably, most reports are observational, and most randomized controlled trials reported a high or unclear risk of bias.

## Conclusion

According to our systematic review, available evidence comparing the transdermal and oral administration routes for HRT is limited. Most studies are observational, and the majority of randomized controlled trials present a high or medium risk of bias. In this scenario, available literature comparing the oral and transdermal administration routes for HRT provides clear evidence only for the VTE risk, which is higher with the oral administration route. Conversely, oral and transdermal administration routes do not appear different regarding an improvement of BMD, glucose metabolism, and lipid profile changes, as well as they do not appear different regarding the risk of breast cancer, endometrial disease, or cardiovascular risk. Considering that the effect on VTE can be considered the clearest and strongest clinical difference between the two administration routes, our systematic review supports the transdermal HRT as safer than the oral administration route. Nevertheless, the final choice of the type of therapy must be tailored and discussed with the patient according to baseline risks and her preferences. Further larger and well-designed studies are mandatory to provide evidence able to guide the personalized choice of HRT.

## Supplementary Information

Below is the link to the electronic supplementary material.

Supplementary file 1 Table S1. Risk of bias assessment of randomized controlled trials (Cochrane Risk of Bias tool). Table S2. Risk of bias assessment of observational studies (The Newcastle-Ottawa Scale for assessing the quality of studies in systematic reviews) (DOCX 34 KB)
