# Supplementary material for: Effects of transdermal versus oral hormone replacement therapy in postmenopause: a systematic review
Source: Arch Gynecol Obstet. 2022 Jun 17;307(6):1727–45. doi: 10.1007/s00404-022-06647-5 (PMC10147786; doi:10.1007/s00404-022-06647-5)
Supplement: Supplementary file 1 — Table S1. Risk of bias assessment of randomized controlled trials (Cochrane Risk of Bias tool). Table S2. Risk of bias assessment of observational studies (The Newcastle-Ottawa Scale for assessing the quality of studies in systematic reviews) (DOCX 34 KB) [file 404_2022_6647_MOESM1_ESM.docx]

| **Supplementary table 1.** Risk of bias assesment of randomized controlled trials (Cochrane Risk of Bias tool) | | | | | | | |
| --- | --- | --- | --- | --- | --- | --- | --- |
| **Author, Year** | **Random Sequence Generation** | **Allocation Concealment** | **Blinding of Participants and Personnel** | **Blinding of Outcome Assessment** | **Incomplete Outcome Data** | **Selective Reporting** | **Overall Risk of Bias** |
| **Perrone G et al., 1996** | Unclear | Unclear | Low | Low | High | Low | Unclear |
| **Adami S et al., 1993** | Low | Unclear | Unclear | Unclear | Unclear | Low | Unclear |
| **Whitcroft SI et al., 1994** | Low | Unclear | Low | Unclear | Unclear | Low | Unclear |
| **Spencer C et al., 1999** | Low | Low | High | Low | Low | Low | High |
| **Erneus M et al., 2001** | Unclear | Unclear | High | High | Low | Low | High |
| **Wakatsuki A et al., 2002** | Low | Unclear | High | High | Low | Low | High |
| **Nanda S et al., 2003** | Low | Unclear | High | High | Low | Low | High |
| **Sanada M et al., 2004** | Low | Unclear | High | High | Low | Low | High |
| **Shakir YA et al. 2004** | Low | Unclear | High | High | Low | Low | High |
| **Vrablik M et al., 2008** | High | Unclear | Unclear | Unclear | Low | Unclear | High |
| **Godsland IF et al., 1993** | Unclear | Unclear | Unclear | Unclear | Low | Low | Unclear |
| **OʼSullivan A et al., 1998** | Unclear | Unclear | High | Low | High | Low | Unclear |
| **Karjalainen A et al., 2001** | Low | Unclear | Low | Low | Low | Low | Low |
| **dos Reis CM et al., 2003** | Low | Low | High | High | Low | Low | High |
| **Chu CM et al., 2006** | Low | Unclear | High | High | Low | Low | High |
| **Russu M et al., 2015** | Unclear | Unclear | High | High | Low | Low | High |
| **Mattsson LA et al. 1999** | High | Unclear | Unclear | Unclear | Unclear | Unclear | High |
| **Sendag F al., 2001** | High | High | Unclear | Unclear | Low | Low | High |
| **Samsioe G et al., 2007** | Unclear | Unclear | High | High | Low | Unclear | High |
| **Stevenson JC et al. 1990** | Low | Low | Low | High | High | Unclear | High |
| **Palacios S et al., 1994** | Unclear | Unclear | Unclear | Unclear | Low | Low | Unclear |
| **Cetinkaya MB et al., 2002** | Low | Unclear | High | High | Low | Low | High |
| **Collete J et al., 2003** | Low | Unclear | Unclear | Unclear | High | Low | Unclear |
| **Davas I et al., 2003** | Unclear | Unclear | High | High | Low | Low | High |

| **Supplementary table 2.** Risk of bias assessment of observational studies (The Newcastle-Ottawa Scale for assessing the quality of studies in systematic review) | | | | | | | | | |
| --- | --- | --- | --- | --- | --- | --- | --- | --- | --- |
| **Studies** | **Selection** | | | | **Comparability** | | **Exposure** | | **Total Quality Score** |
| **Author, year** | **Is the Case Definition Adequate?** | **Representativeness of the Cases** | **Selection of Controls** | **Definition of Controls** | **Comparability of cases and controls** | **Ascertainment of exposure** | **Same method of ascertainment for cases and controls** | **Non-Response Rate** |  |
| **Varas Lorenzo et al., 2000** | 1 | 1 | 1 | 1 | 1 | 1 | 1 | 0 | 7 |
| **Chilvers ECD et al., 2003** | 1 | 1 | 0 | 0 | 1 | 1 | 1 | 1 | 6 |
| **Hippisley-Cox J et al., 2003** | 1 | 1 | 0 | 1 | 1 | 1 | 1 | 1 | 7 |
| **de Vries CS et al., 2006** | 1 | 1 | 1 | 1 | 1 | 1 | 1 | 1 | 8 |
| **Corrao G et al., 2007** | 1 | 1 | 0 | 0 | 1 | 1 | 1 | 1 | 6 |
| **Lokkegaard E et al., 2008** | 1 | 1 | 0 | 1 | 1 | 1 | 1 | 1 | 7 |
| **Daly et al., 1996** | 1 | 1 | 0 | 1 | 1 | 1 | 1 | 1 | 7 |
| **Perez Gutthann S et al., 1997** | 1 | 1 | 0 | 1 | 1 | 1 | 1 | 1 | 7 |
| **Scarabin PY et al., 2003** | 1 | 1 | 0 | 1 | 1 | 1 | 1 | 1 | 7 |
| **Canonico M et al., 2007** | 1 | 1 | 1 | 1 | 1 | 1 | 1 | 0 | 7 |
| **Canonico M et al., 2010** | 1 | 1 | 1 | 1 | 1 | 1 | 1 | 1 | 8 |
| **Renoux S et al., 2010** | 1 | 1 | 0 | 1 | 1 | 1 | 1 | 1 | 7 |
| **Sweetland S et al., 2012** | 1 | 1 | 1 | 1 | 1 | 1 | 1 | 1 | 8 |
| **Vinogradova et al., 2019** | 1 | 1 | 1 | 1 | 1 | 1 | 1 | 1 | 8 |
| **Bergendal et al., 2016** | 1 | 1 | 1 | 1 | 1 | 1 | 1 | 1 | 8 |
| **Simon et al., 2016** | 1 | 1 | 0 | 1 | 1 | 1 | 1 | 1 | 7 |
| **Lee JY et al., 2015** | 1 | 1 | 0 | 1 | 1 | 1 | 1 | 1 | 7 |
| **de Lauzon-Guillain B et al., 2009** | 1 | 1 | 0 | 1 | 1 | 1 | 1 | 1 | 7 |
| **Weiderpass E et al., 1999** | 1 | 1 | 1 | 1 | 1 | 1 | 1 | 1 | 8 |
| **Beral V et al., 2003** | 1 | 1 | 1 | 1 | 1 | 1 | 1 | 1 | 8 |
| **Fournier A et al., 2005** | 1 | 1 | 1 | 1 | 1 | 1 | 1 | 1 | 8 |
| **Lyytinen H et al., 2006** | 1 | 1 | 1 | 1 | 1 | 1 | 1 | 1 | 8 |
| **Lyytinen H et al., 2009** | 1 | 1 | 1 | 1 | 1 | 1 | 1 | 0 | 7 |
| **Fournier A et al., 2008** | 1 | 1 | 1 | 1 | 1 | 1 | 1 | 1 | 8 |
| **Opatrny S et al., 2008** | 1 | 1 | 0 | 1 | 1 | 1 | 1 | 1 | 7 |
| **Corrao G et al., 2008** | 1 | 1 | 0 | 1 | 1 | 1 | 1 | 1 | 7 |
| **Kim H et al., 2014** | 1 | 1 | 0 | 1 | 1 | 1 | 1 | 0 | 6 |
